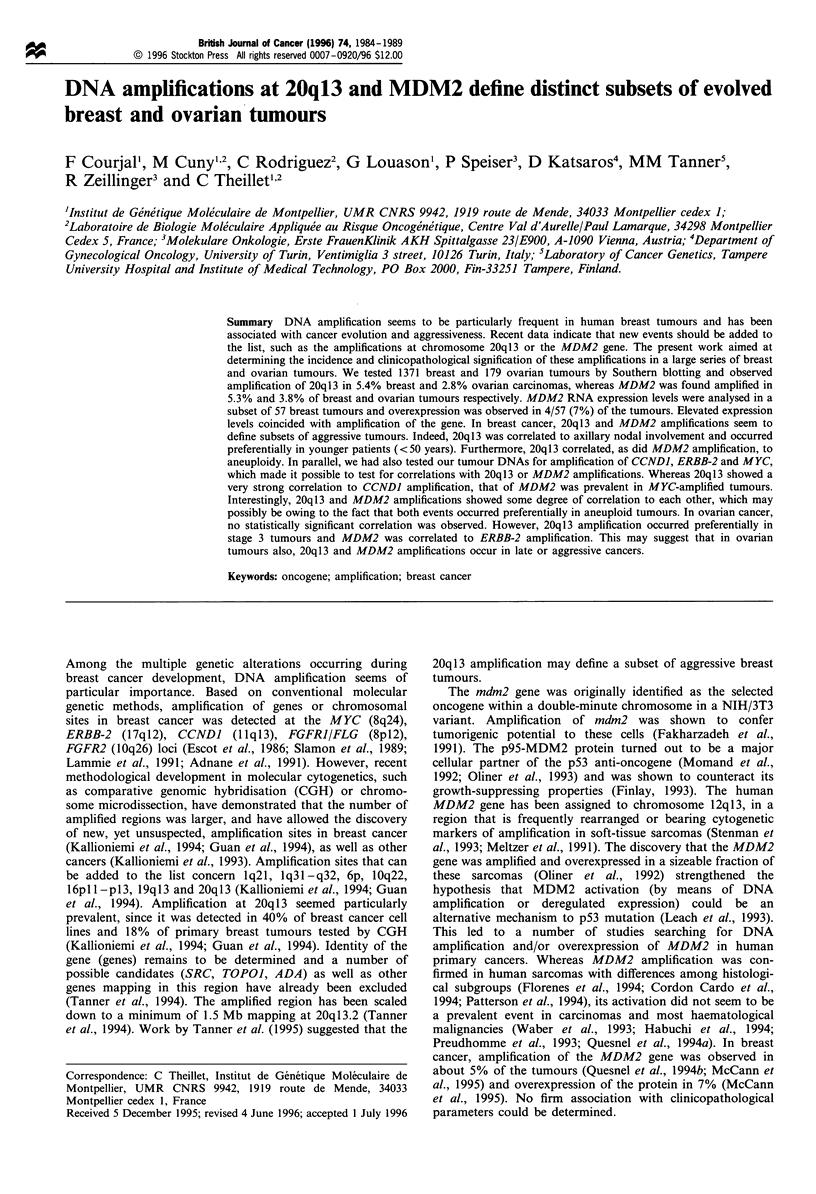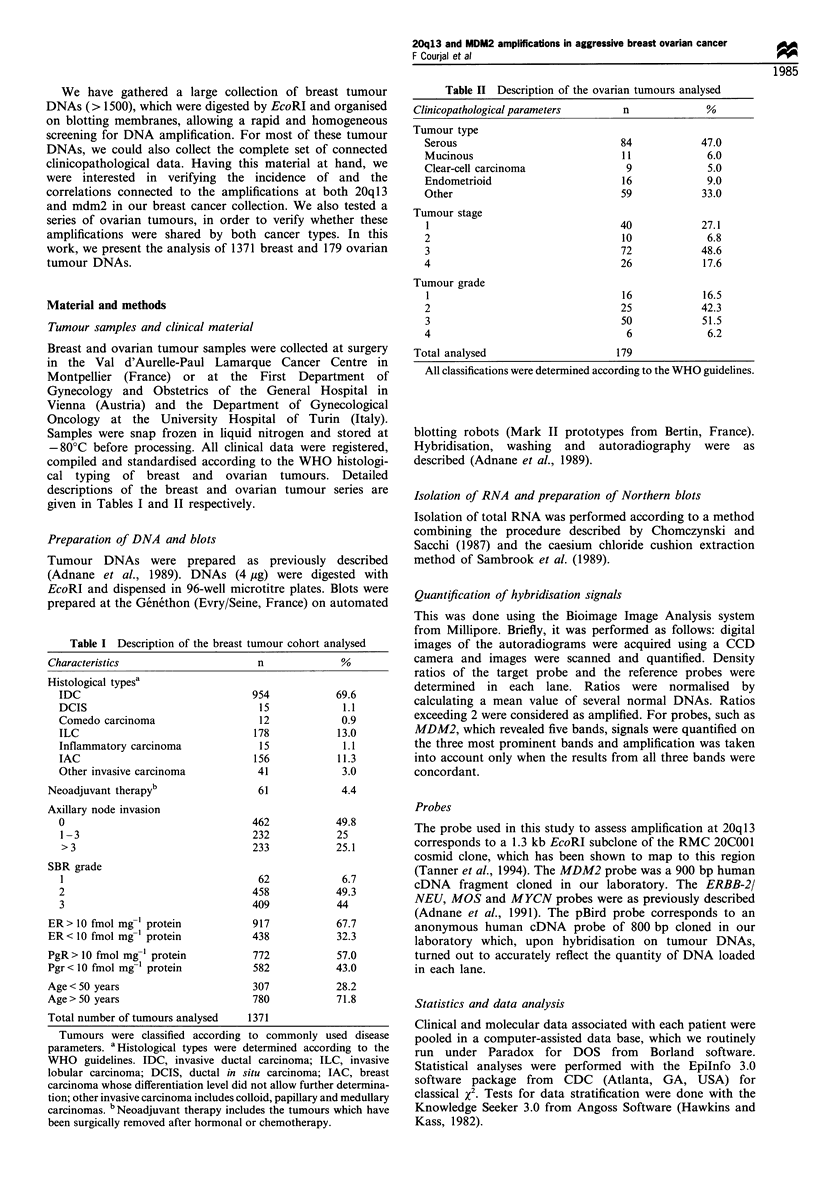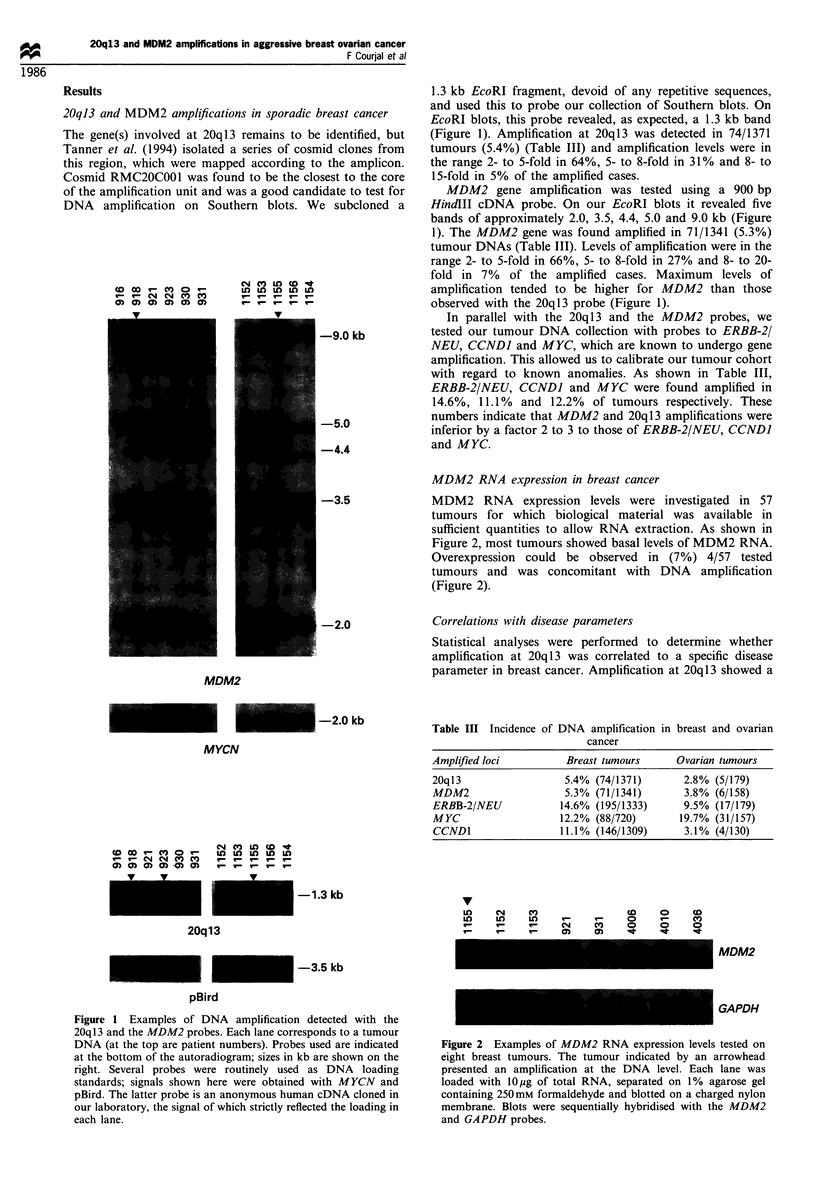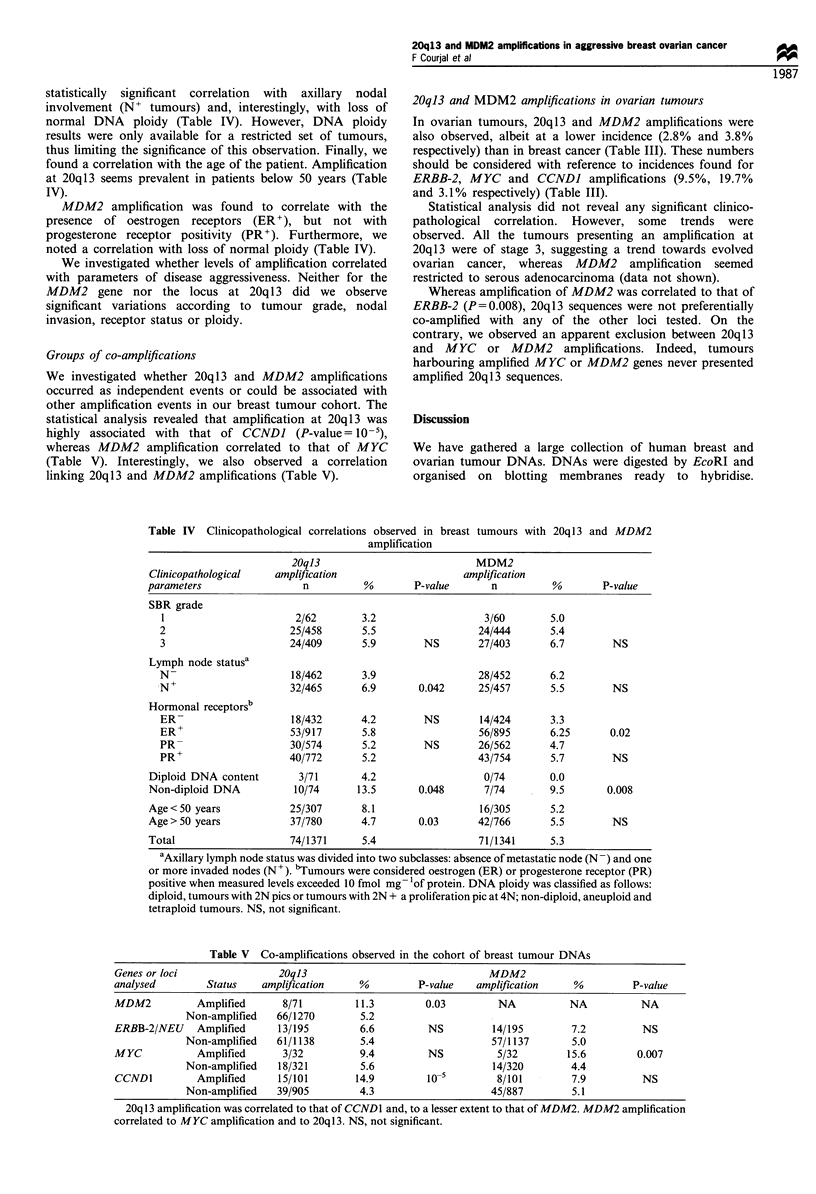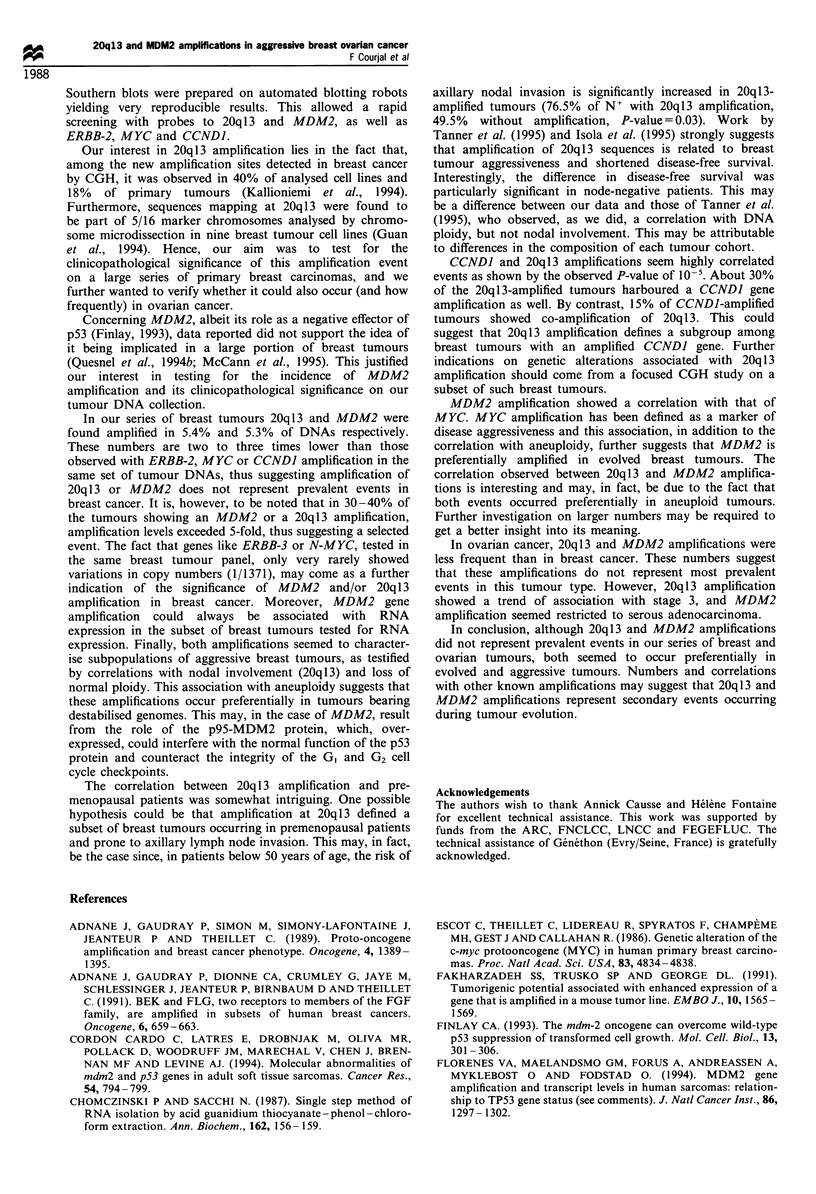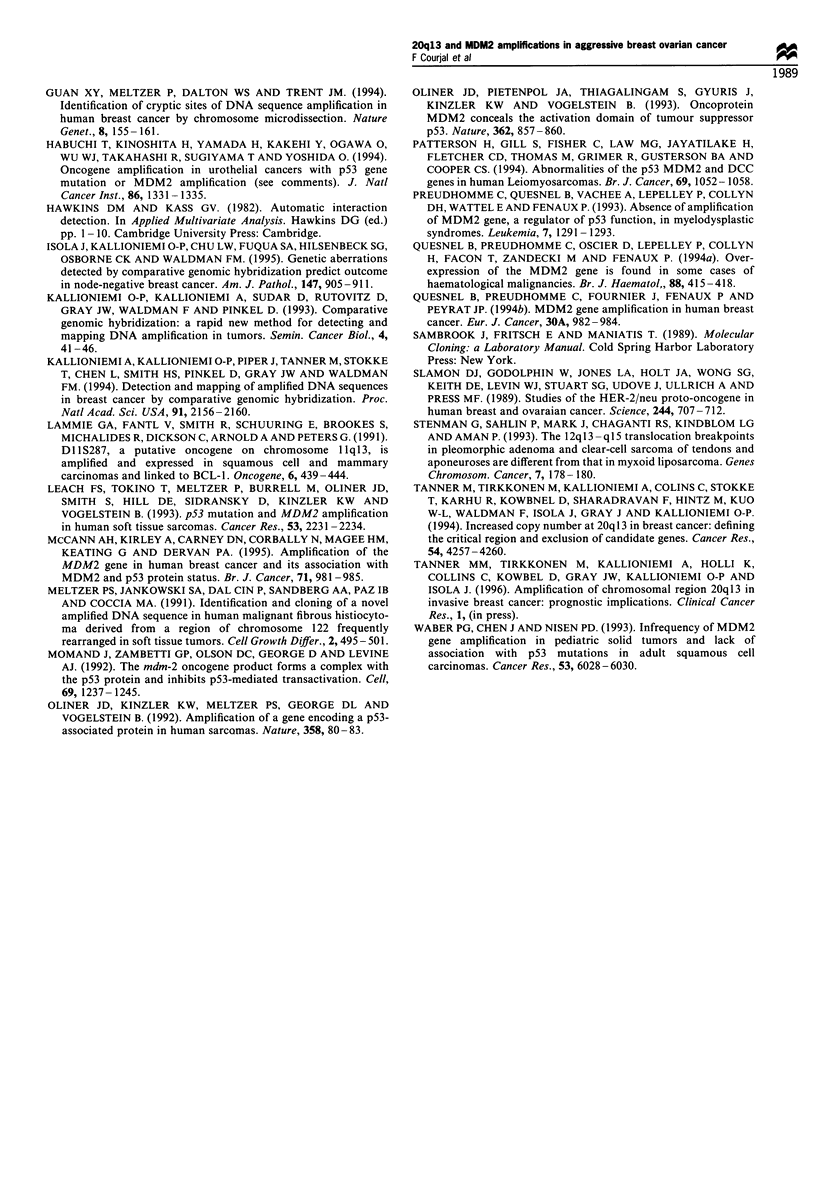# DNA amplifications at 20q13 and MDM2 define distinct subsets of evolved breast and ovarian tumours.

**DOI:** 10.1038/bjc.1996.664

**Published:** 1996-12

**Authors:** F. Courjal, M. Cuny, C. Rodriguez, G. Louason, P. Speiser, D. Katsaros, M. M. Tanner, R. Zeillinger, C. Theillet

**Affiliations:** Institut de Génétique Moléculaire de Montpellier, UMR CNRS 9942, France.

## Abstract

**Images:**


British Journal of Cancer (1996) 74, 1984-1989
© 1996 Stockton Press All rights reserved 0007-0920/96 $12.00

### DNA amplifications at 20q13 and MDM2 define distinct subsets of evolved breast and ovarian tumours

F Courjal[1], M Cuny[1,2], C Rodriguez[2], G Louason[1], P Speiser[3], D Katsaros[4], MM Tanner[5], R Zeillinger[3] and C Theillet[1,2]

[1]*Institut de Génétique Moléculaire de Montpellier, UMR CNRS 9942, 1919 route de Mende, 34033 Montpellier cedex 1;* [2]*Laboratoire de Biologie Moléculaire Appliquée au Risque Oncogénétique, Centre Val d'Aurelle/Paul Lamarque, 34298 Montpellier Cedex 5, France;* [3]*Molekulare Onkologie, Erste FrauenKlinik AKH Spittalgasse 23/E900, A-1090 Vienna, Austria;* [4]*Department of Gynecological Oncology, University of Turin, Ventimiglia 3 street, 10126 Turin, Italy;* [5]*Laboratory of Cancer Genetics, Tampere University Hospital and Institute of Medical Technology, PO Box 2000, Fin-33251 Tampere, Finland.*

**Summary** DNA amplification seems to be particularly frequent in human breast tumours and has been associated with cancer evolution and aggressiveness. Recent data indicate that new events should be added to the list, such as the amplifications at chromosome 20q13 or the *MDM2* gene. The present work aimed at determining the incidence and clinicopathological signification of these amplifications in a large series of breast and ovarian tumours. We tested 1371 breast and 179 ovarian tumours by Southern blotting and observed amplification of 20q13 in 5.4% breast and 2.8% ovarian carcinomas, whereas *MDM2* was found amplified in 5.3% and 3.8% of breast and ovarian tumours respectively. *MDM2* RNA expression levels were analysed in a subset of 57 breast tumours and overexpression was observed in 4/57 (7%) of the tumours. Elevated expression levels coincided with amplification of the gene. In breast cancer, 20q13 and *MDM2* amplifications seem to define subsets of aggressive tumours. Indeed, 20q13 was correlated to axillary nodal involvement and occurred preferentially in younger patients (<50 years). Furthermore, 20q13 correlated, as did *MDM2* amplification, to aneuploidy. In parallel, we had also tested our tumour DNAs for amplification of *CCND1*, *ERBB-2* and *MYC*, which made it possible to test for correlations with 20q13 or *MDM2* amplifications. Whereas 20q13 showed a very strong correlation to *CCND1* amplification, that of *MDM2* was prevalent in *MYC*-amplified tumours. Interestingly, 20q13 and *MDM2* amplifications showed some degree of correlation to each other, which may possibly be owing to the fact that both events occurred preferentially in aneuploid tumours. In ovarian cancer, no statistically significant correlation was observed. However, 20q13 amplification occurred preferentially in stage 3 tumours and *MDM2* was correlated to *ERBB-2* amplification. This may suggest that in ovarian tumours also, 20q13 and *MDM2* amplifications occur in late or aggressive cancers.

**Keywords:** oncogene; amplification; breast cancer

Among the multiple genetic alterations occurring during breast cancer development, DNA amplification seems of particular importance. Based on conventional molecular genetic methods, amplification of genes or chromosomal sites in breast cancer was detected at the *MYC* (8q24), *ERBB-2* (17q12), *CCND1* (11q13), *FGFR1/FLG* (8p12), *FGFR2* (10q26) loci (Escot *et al*., 1986; Slamon *et al*., 1989; Lammie *et al*., 1991; Adnane *et al*., 1991). However, recent methodological development in molecular cytogenetics, such as comparative genomic hybridisation (CGH) or chromosome microdissection, have demonstrated that the number of amplified regions was larger, and have allowed the discovery of new, yet unsuspected, amplification sites in breast cancer (Kallioniemi *et al*., 1994; Guan *et al*., 1994), as well as other cancers (Kallioniemi *et al*., 1993). Amplification sites that can be added to the list concern 1q21, 1q31-q32, 6p, 10q22, 16p11-p13, 19q13 and 20q13 (Kallioniemi *et al*., 1994; Guan *et al*., 1994). Amplification at 20q13 seemed particularly prevalent, since it was detected in 40% of breast cancer cell lines and 18% of primary breast tumours tested by CGH (Kallioniemi *et al*., 1994; Guan *et al*., 1994). Identity of the gene (genes) remains to be determined and a number of possible candidates (*SRC*, *TOPO1*, *ADA*) as well as other genes mapping in this region have already been excluded (Tanner *et al*., 1994). The amplified region has been scaled down to a minimum of 1.5 Mb mapping at 20q13.2 (Tanner *et al*., 1994). Work by Tanner *et al*. (1995) suggested that the 20q13 amplification may define a subset of aggressive breast tumours.

The *mdm2* gene was originally identified as the selected oncogene within a double-minute chromosome in a NIH/3T3 variant. Amplification of *mdm2* was shown to confer tumorigenic potential to these cells (Fakharzadeh *et al*., 1991). The p95-MDM2 protein turned out to be a major cellular partner of the p53 anti-oncogene (Momand *et al*., 1992; Oliner *et al*., 1993) and was shown to counteract its growth-suppressing properties (Finlay, 1993). The human *MDM2* gene has been assigned to chromosome 12q13, in a region that is frequently rearranged or bearing cytogenetic markers of amplification in soft-tissue sarcomas (Stenman *et al*., 1993; Meltzer *et al*., 1991). The discovery that the *MDM2* gene was amplified and overexpressed in a sizeable fraction of these sarcomas (Oliner *et al*., 1992) strengthened the hypothesis that MDM2 activation (by means of DNA amplification or deregulated expression) could be an alternative mechanism to p53 mutation (Leach *et al*., 1993). This led to a number of studies searching for DNA amplification and/or overexpression of *MDM2* in human primary cancers. Whereas *MDM2* amplification was confirmed in human sarcomas with differences among histological subgroups (Florenes *et al*., 1994; Cordon Cardo *et al*., 1994; Patterson *et al*., 1994), its activation did not seem to be a prevalent event in carcinomas and most haematological malignancies (Waber *et al*., 1993; Habuchi *et al*., 1994; Preudhomme *et al*., 1993; Quesnel *et al*., 1994*a*). In breast cancer, amplification of the *MDM2* gene was observed in about 5% of the tumours (Quesnel *et al*., 1994*b*; McCann *et al*., 1995) and overexpression of the protein in 7% (McCann *et al*., 1995). No firm association with clinicopathological parameters could be determined.

Correspondence: C Theillet, Institut de Génétique Moléculaire de Montpellier, UMR CNRS 9942, 1919 route de Mende, 34033 Montpellier cedex 1, France
Received 5 December 1995; revised 4 June 1996; accepted 1 July 1996

We have gathered a large collection of breast tumour DNAs (> 1500), which were digested by *Eco*RI and organised on blotting membranes, allowing a rapid and homogeneous screening for DNA amplification. For most of these tumour DNAs, we could also collect the complete set of connected clinicopathological data. Having this material at hand, we were interested in verifying the incidence of and the correlations connected to the amplifications at both 20q13 and mdm2 in our breast cancer collection. We also tested a series of ovarian tumours, in order to verify whether these amplifications were shared by both cancer types. In this work, we present the analysis of 1371 breast and 179 ovarian tumour DNAs.

### Material and methods

### *Tumour samples and clinical material*

Breast and ovarian tumour samples were collected at surgery in the Val d'Aurelle-Paul Lamarque Cancer Centre in Montpellier (France) or at the First Department of Gynecology and Obstetrics of the General Hospital in Vienna (Austria) and the Department of Gynecological Oncology at the University Hospital of Turin (Italy). Samples were snap frozen in liquid nitrogen and stored at −80°C before processing. All clinical data were registered, compiled and standardised according to the WHO histological typing of breast and ovarian tumours. Detailed descriptions of the breast and ovarian tumour series are given in Tables I and II respectively.

### *Preparation of DNA and blots*

Tumour DNAs were prepared as previously described (Adnane *et al.*, 1989). DNAs (4 μg) were digested with *Eco*RI and dispensed in 96-well microtitre plates. Blots were prepared at the Généthon (Evry/Seine, France) on automated blotting robots (Mark II prototypes from Bertin, France). Hybridisation, washing and autoradiography were as described (Adnane *et al.*, 1989).

**Table I** Description of the breast tumour cohort analysed

| *Characteristics* | n | % |
|---|---|---|
| Histological types[a] | | |
| IDC | 954 | 69.6 |
| DCIS | 15 | 1.1 |
| Comedo carcinoma | 12 | 0.9 |
| ILC | 178 | 13.0 |
| Inflammatory carcinoma | 15 | 1.1 |
| IAC | 156 | 11.3 |
| Other invasive carcinoma | 41 | 3.0 |
| Neoadjuvant therapy[b] | 61 | 4.4 |
| Axillary node invasion | | |
| 0 | 462 | 49.8 |
| 1–3 | 232 | 25 |
| >3 | 233 | 25.1 |
| SBR grade | | |
| 1 | 62 | 6.7 |
| 2 | 458 | 49.3 |
| 3 | 409 | 44 |
| ER > 10 fmol $mg^{-1}$ protein | 917 | 67.7 |
| ER < 10 fmol $mg^{-1}$ protein | 438 | 32.3 |
| PgR > 10 fmol $mg^{-1}$ protein | 772 | 57.0 |
| Pgr < 10 fmol $mg^{-1}$ protein | 582 | 43.0 |
| Age < 50 years | 307 | 28.2 |
| Age > 50 years | 780 | 71.8 |
| Total number of tumours analysed | 1371 | |

Tumours were classified according to commonly used disease parameters. [a] Histological types were determined according to the WHO guidelines. IDC, invasive ductal carcinoma; ILC, invasive lobular carcinoma; DCIS, ductal *in situ* carcinoma; IAC, breast carcinoma whose differentiation level did not allow further determination; other invasive carcinoma includes colloid, papillary and medullary carcinomas. [b] Neoadjuvant therapy includes the tumours which have been surgically removed after hormonal or chemotherapy.

**Table II** Description of the ovarian tumours analysed

| *Clinicopathological parameters* | n | % |
|---|---|---|
| Tumour type | | |
| Serous | 84 | 47.0 |
| Mucinous | 11 | 6.0 |
| Clear-cell carcinoma | 9 | 5.0 |
| Endometrioid | 16 | 9.0 |
| Other | 59 | 33.0 |
| Tumour stage | | |
| 1 | 40 | 27.1 |
| 2 | 10 | 6.8 |
| 3 | 72 | 48.6 |
| 4 | 26 | 17.6 |
| Tumour grade | | |
| 1 | 16 | 16.5 |
| 2 | 25 | 42.3 |
| 3 | 50 | 51.5 |
| 4 | 6 | 6.2 |
| Total analysed | 179 | |

All classifications were determined according to the WHO guidelines.

### *Isolation of RNA and preparation of Northern blots*

Isolation of total RNA was performed according to a method combining the procedure described by Chomczynski and Sacchi (1987) and the caesium chloride cushion extraction method of Sambrook *et al.* (1989).

### *Quantification of hybridisation signals*

This was done using the Bioimage Image Analysis system from Millipore. Briefly, it was performed as follows: digital images of the autoradiograms were acquired using a CCD camera and images were scanned and quantified. Density ratios of the target probe and the reference probes were determined in each lane. Ratios were normalised by calculating a mean value of several normal DNAs. Ratios exceeding 2 were considered as amplified. For probes, such as *MDM2*, which revealed five bands, signals were quantified on the three most prominent bands and amplification was taken into account only when the results from all three bands were concordant.

### *Probes*

The probe used in this study to assess amplification at 20q13 corresponds to a 1.3 kb *Eco*RI subclone of the RMC 20C001 cosmid clone, which has been shown to map to this region (Tanner *et al.*, 1994). The *MDM2* probe was a 900 bp human cDNA fragment cloned in our laboratory. The *ERBB-2/NEU*, *MOS* and *MYCN* probes were as previously described (Adnane *et al.*, 1991). The pBird probe corresponds to an anonymous human cDNA probe of 800 bp cloned in our laboratory which, upon hybridisation on tumour DNAs, turned out to accurately reflect the quantity of DNA loaded in each lane.

### *Statistics and data analysis*

Clinical and molecular data associated with each patient were pooled in a computer-assisted data base, which we routinely run under Paradox for DOS from Borland software. Statistical analyses were performed with the EpiInfo 3.0 software package from CDC (Atlanta, GA, USA) for classical $\chi^2$. Tests for data stratification were done with the Knowledge Seeker 3.0 from Angoss Software (Hawkins and Kass, 1982).

### Results

### *20q13 and* MDM2 *amplifications in sporadic breast cancer*

The gene(s) involved at 20q13 remains to be identified, but Tanner *et al.* (1994) isolated a series of cosmid clones from this region, which were mapped according to the amplicon. Cosmid RMC20C001 was found to be the closest to the core of the amplification unit and was a good candidate to test for DNA amplification on Southern blots. We subcloned a 1.3 kb *Eco*RI fragment, devoid of any repetitive sequences, and used this to probe our collection of Southern blots. On *Eco*RI blots, this probe revealed, as expected, a 1.3 kb band (Figure 1). Amplification at 20q13 was detected in 74/1371 tumours (5.4%) (Table III) and amplification levels were in the range 2- to 5-fold in 64%, 5- to 8-fold in 31% and 8- to 15-fold in 5% of the amplified cases.

*MDM2* gene amplification was tested using a 900 bp *Hind*III cDNA probe. On our *Eco*RI blots it revealed five bands of approximately 2.0, 3.5, 4.4, 5.0 and 9.0 kb (Figure 1). The *MDM2* gene was found amplified in 71/1341 (5.3%) tumour DNAs (Table III). Levels of amplification were in the range 2- to 5-fold in 66%, 5- to 8-fold in 27% and 8- to 20-fold in 7% of the amplified cases. Maximum levels of amplification tended to be higher for *MDM2* than those observed with the 20q13 probe (Figure 1).

In parallel with the 20q13 and the *MDM2* probes, we tested our tumour DNA collection with probes to *ERBB-2/NEU*, *CCND1* and *MYC*, which are known to undergo gene amplification. This allowed us to calibrate our tumour cohort with regard to known anomalies. As shown in Table III, *ERBB-2/NEU*, *CCND1* and *MYC* were found amplified in 14.6%, 11.1% and 12.2% of tumours respectively. These numbers indicate that *MDM2* and 20q13 amplifications were inferior by a factor 2 to 3 to those of *ERBB-2/NEU*, *CCND1* and *MYC*.

**Figure 1** Examples of DNA amplification detected with the 20q13 and the *MDM2* probes. Each lane corresponds to a tumour DNA (at the top are patient numbers). Probes used are indicated at the bottom of the autoradiogram; sizes in kb are shown on the right. Several probes were routinely used as DNA loading standards; signals shown here were obtained with *MYCN* and pBird. The latter probe is an anonymous human cDNA cloned in our laboratory, the signal of which strictly reflected the loading in each lane.

### *MDM2 RNA expression in breast cancer*

MDM2 RNA expression levels were investigated in 57 tumours for which biological material was available in sufficient quantities to allow RNA extraction. As shown in Figure 2, most tumours showed basal levels of MDM2 RNA. Overexpression could be observed in (7%) 4/57 tested tumours and was concomitant with DNA amplification (Figure 2).

### *Correlations with disease parameters*

Statistical analyses were performed to determine whether amplification at 20q13 was correlated to a specific disease parameter in breast cancer. Amplification at 20q13 showed a

**Table III** Incidence of DNA amplification in breast and ovarian cancer

| *Amplified loci* | *Breast tumours* | *Ovarian tumours* |
|---|---|---|
| 20q13 | 5.4% (74/1371) | 2.8% (5/179) |
| *MDM*2 | 5.3% (71/1341) | 3.8% (6/158) |
| *ERB*B-2/*NEU* | 14.6% (195/1333) | 9.5% (17/179) |
| *MYC* | 12.2% (88/720) | 19.7% (31/157) |
| *CCND*1 | 11.1% (146/1309) | 3.1% (4/130) |

**Figure 2** Examples of *MDM2* RNA expression levels tested on eight breast tumours. The tumour indicated by an arrowhead presented an amplification at the DNA level. Each lane was loaded with 10 μg of total RNA, separated on 1% agarose gel containing 250 mM formaldehyde and blotted on a charged nylon membrane. Blots were sequentially hybridised with the *MDM2* and *GAPDH* probes.

statistically significant correlation with axillary nodal involvement ($N^+$ tumours) and, interestingly, with loss of normal DNA ploidy (Table IV). However, DNA ploidy results were only available for a restricted set of tumours, thus limiting the significance of this observation. Finally, we found a correlation with the age of the patient. Amplification at 20q13 seems prevalent in patients below 50 years (Table IV).

*MDM2* amplification was found to correlate with the presence of oestrogen receptors ($ER^+$), but not with progesterone receptor positivity ($PR^+$). Furthermore, we noted a correlation with loss of normal ploidy (Table IV).

We investigated whether levels of amplification correlated with parameters of disease aggressiveness. Neither for the *MDM2* gene nor the locus at 20q13 did we observe significant variations according to tumour grade, nodal invasion, receptor status or ploidy.

### *Groups of co-amplifications*

We investigated whether 20q13 and *MDM2* amplifications occurred as independent events or could be associated with other amplification events in our breast tumour cohort. The statistical analysis revealed that amplification at 20q13 was highly associated with that of *CCND1* ($P$-value$=10^{-5}$), whereas *MDM2* amplification correlated to that of *MYC* (Table V). Interestingly, we also observed a correlation linking 20q13 and *MDM2* amplifications (Table V).

### *20q13 and* MDM2 *amplifications in ovarian tumours*

In ovarian tumours, 20q13 and *MDM2* amplifications were also observed, albeit at a lower incidence (2.8% and 3.8% respectively) than in breast cancer (Table III). These numbers should be considered with reference to incidences found for *ERBB-2*, *MYC* and *CCND1* amplifications (9.5%, 19.7% and 3.1% respectively) (Table III).

Statistical analysis did not reveal any significant clinicopathological correlation. However, some trends were observed. All the tumours presenting an amplification at 20q13 were of stage 3, suggesting a trend towards evolved ovarian cancer, whereas *MDM2* amplification seemed restricted to serous adenocarcinoma (data not shown).

Whereas amplification of *MDM2* was correlated to that of *ERBB-2* ($P=0.008$), 20q13 sequences were not preferentially co-amplified with any of the other loci tested. On the contrary, we observed an apparent exclusion between 20q13 and *MYC* or *MDM2* amplifications. Indeed, tumours harbouring amplified *MYC* or *MDM2* genes never presented amplified 20q13 sequences.

### Discussion

We have gathered a large collection of human breast and ovarian tumour DNAs. DNAs were digested by *Eco*RI and organised on blotting membranes ready to hybridise.

**Table IV** Clinicopathological correlations observed in breast tumours with 20q13 and *MDM2* amplification

| *Clinicopathological parameters* | *20q13 amplification* n | % | P-*value* | MDM2 *amplification* n | % | P-*value* |
|---|---|---|---|---|---|---|
| SBR grade | | | | | | |
| 1 | 2/62 | 3.2 | | 3/60 | 5.0 | |
| 2 | 25/458 | 5.5 | | 24/444 | 5.4 | |
| 3 | 24/409 | 5.9 | NS | 27/403 | 6.7 | NS |
| Lymph node status[a] | | | | | | |
| $N^-$ | 18/462 | 3.9 | | 28/452 | 6.2 | |
| $N^+$ | 32/465 | 6.9 | 0.042 | 25/457 | 5.5 | NS |
| Hormonal receptors[b] | | | | | | |
| $ER^-$ | 18/432 | 4.2 | NS | 14/424 | 3.3 | |
| $ER^+$ | 53/917 | 5.8 | | 56/895 | 6.25 | 0.02 |
| $PR^-$ | 30/574 | 5.2 | NS | 26/562 | 4.7 | |
| $PR^+$ | 40/772 | 5.2 | | 43/754 | 5.7 | NS |
| Diploid DNA content | 3/71 | 4.2 | | 0/74 | 0.0 | |
| Non-diploid DNA | 10/74 | 13.5 | 0.048 | 7/74 | 9.5 | 0.008 |
| Age < 50 years | 25/307 | 8.1 | | 16/305 | 5.2 | |
| Age > 50 years | 37/780 | 4.7 | 0.03 | 42/766 | 5.5 | NS |
| Total | 74/1371 | 5.4 | | 71/1341 | 5.3 | |

[a]Axillary lymph node status was divided into two subclasses: absence of metastatic node ($N^-$) and one or more invaded nodes ($N^+$). [b]Tumours were considered oestrogen (ER) or progesterone receptor (PR) positive when measured levels exceeded 10 fmol $mg^{-1}$of protein. DNA ploidy was classified as follows: diploid, tumours with 2N pics or tumours with 2N + a proliferation pic at 4N; non-diploid, aneuploid and tetraploid tumours. NS, not significant.

**Table V** Co-amplifications observed in the cohort of breast tumour DNAs

| *Genes or loci analysed* | *Status* | *20q13 amplification* | % | P-*value* | *MDM2 amplification* | % | P-*value* |
|---|---|---|---|---|---|---|---|
| *MDM2* | Amplified | 8/71 | 11.3 | 0.03 | NA | NA | NA |
| | Non-amplified | 66/1270 | 5.2 | | | | |
| *ERB*B-2/*NEU* | Amplified | 13/195 | 6.6 | NS | 14/195 | 7.2 | NS |
| | Non-amplified | 61/1138 | 5.4 | | 57/1137 | 5.0 | |
| *MYC* | Amplified | 3/32 | 9.4 | NS | 5/32 | 15.6 | 0.007 |
| | Non-amplified | 18/321 | 5.6 | | 14/320 | 4.4 | |
| *CCND*1 | Amplified | 15/101 | 14.9 | $10^{-5}$ | 8/101 | 7.9 | NS |
| | Non-amplified | 39/905 | 4.3 | | 45/887 | 5.1 | |

20q13 amplification was correlated to that of *CCND*1 and, to a lesser extent to that of *MDM*2. *MDM*2 amplification correlated to *MYC* amplification and to 20q13. NS, not significant.

Southern blots were prepared on automated blotting robots yielding very reproducible results. This allowed a rapid screening with probes to 20q13 and *MDM2*, as well as *ERBB-2*, *MYC* and *CCND1*.

Our interest in 20q13 amplification lies in the fact that, among the new amplification sites detected in breast cancer by CGH, it was observed in 40% of analysed cell lines and 18% of primary tumours (Kallioniemi *et al.*, 1994). Furthermore, sequences mapping at 20q13 were found to be part of 5/16 marker chromosomes analysed by chromosome microdissection in nine breast tumour cell lines (Guan *et al.*, 1994). Hence, our aim was to test for the clinicopathological significance of this amplification event on a large series of primary breast carcinomas, and we further wanted to verify whether it could also occur (and how frequently) in ovarian cancer.

Concerning *MDM2*, albeit its role as a negative effector of p53 (Finlay, 1993), data reported did not support the idea of it being implicated in a large portion of breast tumours (Quesnel *et al.*, 1994*b*; McCann *et al.*, 1995). This justified our interest in testing for the incidence of *MDM2* amplification and its clinicopathological significance on our tumour DNA collection.

In our series of breast tumours 20q13 and *MDM2* were found amplified in 5.4% and 5.3% of DNAs respectively. These numbers are two to three times lower than those observed with *ERBB-2*, *MYC* or *CCND1* amplification in the same set of tumour DNAs, thus suggesting amplification of 20q13 or *MDM2* does not represent prevalent events in breast cancer. It is, however, to be noted that in 30–40% of the tumours showing an *MDM2* or a 20q13 amplification, amplification levels exceeded 5-fold, thus suggesting a selected event. The fact that genes like *ERBB-3* or *N-MYC*, tested in the same breast tumour panel, only very rarely showed variations in copy numbers (1/1371), may come as a further indication of the significance of *MDM2* and/or 20q13 amplification in breast cancer. Moreover, *MDM2* gene amplification could always be associated with RNA expression in the subset of breast tumours tested for RNA expression. Finally, both amplifications seemed to characterise subpopulations of aggressive breast tumours, as testified by correlations with nodal involvement (20q13) and loss of normal ploidy. This association with aneuploidy suggests that these amplifications occur preferentially in tumours bearing destabilised genomes. This may, in the case of *MDM2*, result from the role of the p95-MDM2 protein, which, overexpressed, could interfere with the normal function of the p53 protein and counteract the integrity of the $G_1$ and $G_2$ cell cycle checkpoints.

The correlation between 20q13 amplification and premenopausal patients was somewhat intriguing. One possible hypothesis could be that amplification at 20q13 defined a subset of breast tumours occurring in premenopausal patients and prone to axillary lymph node invasion. This may, in fact, be the case since, in patients below 50 years of age, the risk of axillary nodal invasion is significantly increased in 20q13-amplified tumours (76.5% of $N^+$ with 20q13 amplification, 49.5% without amplification, *P*-value = 0.03). Work by Tanner *et al.* (1995) and Isola *et al.* (1995) strongly suggests that amplification of 20q13 sequences is related to breast tumour aggressiveness and shortened disease-free survival. Interestingly, the difference in disease-free survival was particularly significant in node-negative patients. This may be a difference between our data and those of Tanner *et al.* (1995), who observed, as we did, a correlation with DNA ploidy, but not nodal involvement. This may be attributable to differences in the composition of each tumour cohort.

*CCND1* and 20q13 amplifications seem highly correlated events as shown by the observed *P*-value of $10^{-5}$. About 30% of the 20q13-amplified tumours harboured a *CCND1* gene amplification as well. By contrast, 15% of *CCND1*-amplified tumours showed co-amplification of 20q13. This could suggest that 20q13 amplification defines a subgroup among breast tumours with an amplified *CCND1* gene. Further indications on genetic alterations associated with 20q13 amplification should come from a focused CGH study on a subset of such breast tumours.

*MDM2* amplification showed a correlation with that of *MYC*. *MYC* amplification has been defined as a marker of disease aggressiveness and this association, in addition to the correlation with aneuploidy, further suggests that *MDM2* is preferentially amplified in evolved breast tumours. The correlation observed between 20q13 and *MDM2* amplifications is interesting and may, in fact, be due to the fact that both events occurred preferentially in aneuploid tumours. Further investigation on larger numbers may be required to get a better insight into its meaning.

In ovarian cancer, 20q13 and *MDM2* amplifications were less frequent than in breast cancer. These numbers suggest that these amplifications do not represent most prevalent events in this tumour type. However, 20q13 amplification showed a trend of association with stage 3, and *MDM2* amplification seemed restricted to serous adenocarcinoma.

In conclusion, although 20q13 and *MDM2* amplifications did not represent prevalent events in our series of breast and ovarian tumours, both seemed to occur preferentially in evolved and aggressive tumours. Numbers and correlations with other known amplifications may suggest that 20q13 and *MDM2* amplifications represent secondary events occurring during tumour evolution.

**Acknowledgements**
The authors wish to thank Annick Causse and Hélène Fontaine for excellent technical assistance. This work was supported by funds from the ARC, FNCLCC, LNCC and FEGEFLUC. The technical assistance of Généthon (Evry/Seine, France) is gratefully acknowledged.

### References

ADNANE J, GAUDRAY P, SIMON M, SIMONY-LAFONTAINE J, JEANTEUR P AND THEILLET C. (1989). Proto-oncogene amplification and breast cancer phenotype. *Oncogene*, **4**, 1389–1395.

ADNANE J, GAUDRAY P, DIONNE CA, CRUMLEY G, JAYE M, SCHLESSINGER J, JEANTEUR P, BIRNBAUM D AND THEILLET C. (1991). BEK and FLG, two receptors to members of the FGF family, are amplified in subsets of human breast cancers. *Oncogene*, **6**, 659–663.

CORDON CARDO C, LATRES E, DROBNJAK M, OLIVA MR, POLLACK D, WOODRUFF JM, MARECHAL V, CHEN J, BRENNAN MF AND LEVINE AJ. (1994). Molecular abnormalities of *mdm2* and *p53* genes in adult soft tissue sarcomas. *Cancer Res.*, **54**, 794–799.

CHOMCZINSKI P AND SACCHI N. (1987). Single step method of RNA isolation by acid guanidium thiocyanate–phenol–chloroform extraction. *Ann. Biochem.*, **162**, 156–159.

ESCOT C, THEILLET C, LIDEREAU R, SPYRATOS F, CHAMPÈME MH, GEST J AND CALLAHAN R. (1986). Genetic alteration of the c-*myc* protooncogene (MYC) in human primary breast carcinomas. *Proc. Natl Acad. Sci. USA*, **83**, 4834–4838.

FAKHARZADEH SS, TRUSKO SP AND GEORGE DL. (1991). Tumorigenic potential associated with enhanced expression of a gene that is amplified in a mouse tumor line. *EMBO J.*, **10**, 1565–1569.

FINLAY CA. (1993). The *mdm*-2 oncogene can overcome wild-type p53 suppression of transformed cell growth. *Mol. Cell. Biol.*, **13**, 301–306.

FLORENES VA, MAELANDSMO GM, FORUS A, ANDREASSEN A, MYKLEBOST O AND FODSTAD O. (1994). MDM2 gene amplification and transcript levels in human sarcomas: relationship to TP53 gene status (see comments). *J. Natl Cancer Inst.*, **86**, 1297–1302.

GUAN XY, MELTZER P, DALTON WS AND TRENT JM. (1994). Identification of cryptic sites of DNA sequence amplification in human breast cancer by chromosome microdissection. *Nature Genet.*, **8**, 155–161.

HABUCHI T, KINOSHITA H, YAMADA H, KAKEHI Y, OGAWA O, WU WJ, TAKAHASHI R, SUGIYAMA T AND YOSHIDA O. (1994). Oncogene amplification in urothelial cancers with p53 gene mutation or MDM2 amplification (see comments). *J. Natl Cancer Inst.*, **86**, 1331–1335.

HAWKINS DM AND KASS GV. (1982). Automatic interaction detection. In *Applied Multivariate Analysis*. Hawkins DG (ed.) pp. 1–10. Cambridge University Press: Cambridge.

ISOLA J, KALLIONIEMI O-P, CHU LW, FUQUA SA, HILSENBECK SG, OSBORNE CK AND WALDMAN FM. (1995). Genetic aberrations detected by comparative genomic hybridization predict outcome in node-negative breast cancer. *Am. J. Pathol.*, **147**, 905–911.

KALLIONIEMI O-P, KALLIONIEMI A, SUDAR D, RUTOVITZ D, GRAY JW, WALDMAN F AND PINKEL D. (1993). Comparative genomic hybridization: a rapid new method for detecting and mapping DNA amplification in tumors. *Semin. Cancer Biol.*, **4**, 41–46.

KALLIONIEMI A, KALLIONIEMI O-P, PIPER J, TANNER M, STOKKE T, CHEN L, SMITH HS, PINKEL D, GRAY JW AND WALDMAN FM. (1994). Detection and mapping of amplified DNA sequences in breast cancer by comparative genomic hybridization. *Proc. Natl Acad. Sci. USA*, **91**, 2156–2160.

LAMMIE GA, FANTL V, SMITH R, SCHUURING E, BROOKES S, MICHALIDES R, DICKSON C, ARNOLD A AND PETERS G. (1991). D11S287, a putative oncogene on chromosome 11q13, is amplified and expressed in squamous cell and mammary carcinomas and linked to BCL-1. *Oncogene*, **6**, 439–444.

LEACH FS, TOKINO T, MELTZER P, BURRELL M, OLINER JD, SMITH S, HILL DE, SIDRANSKY D, KINZLER KW AND VOGELSTEIN B. (1993). *p53* mutation and *MDM2* amplification in human soft tissue sarcomas. *Cancer Res.*, **53**, 2231–2234.

McCANN AH, KIRLEY A, CARNEY DN, CORBALLY N, MAGEE HM, KEATING G AND DERVAN PA. (1995). Amplification of the *MDM2* gene in human breast cancer and its association with MDM2 and p53 protein status. *Br. J. Cancer*, **71**, 981–985.

MELTZER PS, JANKOWSKI SA, DAL CIN P, SANDBERG AA, PAZ IB AND COCCIA MA. (1991). Identification and cloning of a novel amplified DNA sequence in human malignant fibrous histiocytoma derived from a region of chromosome 122 frequently rearranged in soft tissue tumors. *Cell Growth Differ.*, **2**, 495–501.

MOMAND J, ZAMBETTI GP, OLSON DC, GEORGE D AND LEVINE AJ. (1992). The *mdm*-2 oncogene product forms a complex with the p53 protein and inhibits p53-mediated transactivation. *Cell*, **69**, 1237–1245.

OLINER JD, KINZLER KW, MELTZER PS, GEORGE DL AND VOGELSTEIN B. (1992). Amplification of a gene encoding a p53-associated protein in human sarcomas. *Nature*, **358**, 80–83.

OLINER JD, PIETENPOL JA, THIAGALINGAM S, GYURIS J, KINZLER KW AND VOGELSTEIN B. (1993). Oncoprotein MDM2 conceals the activation domain of tumour suppressor p53. *Nature*, **362**, 857–860.

PATTERSON H, GILL S, FISHER C, LAW MG, JAYATILAKE H, FLETCHER CD, THOMAS M, GRIMER R, GUSTERSON BA AND COOPER CS. (1994). Abnormalities of the p53 MDM2 and DCC genes in human Leiomyosarcomas. *Br. J. Cancer*, **69**, 1052–1058.

PREUDHOMME C, QUESNEL B, VACHEE A, LEPELLEY P, COLLYN DH, WATTEL E AND FENAUX P. (1993). Absence of amplification of MDM2 gene, a regulator of p53 function, in myelodysplastic syndromes. *Leukemia*, **7**, 1291–1293.

QUESNEL B, PREUDHOMME C, OSCIER D, LEPELLEY P, COLLYN H, FACON T, ZANDECKI M AND FENAUX P. (1994*a*). Over-expression of the MDM2 gene is found in some cases of haematological malignancies. *Br. J. Haematol,*, **88**, 415–418.

QUESNEL B, PREUDHOMME C, FOURNIER J, FENAUX P AND PEYRAT JP. (1994*b*). MDM2 gene amplification in human breast cancer. *Eur. J. Cancer*, **30A**, 982–984.

SAMBROOK J, FRITSCH E AND MANIATIS T. (1989). *Molecular Cloning: a Laboratory Manual.* Cold Spring Harbor Laboratory Press: New York.

SLAMON DJ, GODOLPHIN W, JONES LA, HOLT JA, WONG SG, KEITH DE, LEVIN WJ, STUART SG, UDOVE J, ULLRICH A AND PRESS MF. (1989). Studies of the HER-2/neu proto-oncogene in human breast and ovaraian cancer. *Science*, **244**, 707–712.

STENMAN G, SAHLIN P, MARK J, CHAGANTI RS, KINDBLOM LG AND AMAN P. (1993). The 12q13–q15 translocation breakpoints in pleomorphic adenoma and clear-cell sarcoma of tendons and aponeuroses are different from that in myxoid liposarcoma. *Genes Chromosom. Cancer*, **7**, 178–180.

TANNER M, TIRKKONEN M, KALLIONIEMI A, COLINS C, STOKKE T, KARHU R, KOWBNEL D, SHARADRAVAN F, HINTZ M, KUO W-L, WALDMAN F, ISOLA J, GRAY J AND KALLIONIEMI O-P. (1994). Increased copy number at 20q13 in breast cancer: defining the critical region and exclusion of candidate genes. *Cancer Res.*, **54**, 4257–4260.

TANNER MM, TIRKKONEN M, KALLIONIEMI A, HOLLI K, COLLINS C, KOWBEL D, GRAY JW, KALLIONIEMI O-P AND ISOLA J. (1996). Amplification of chromosomal region 20q13 in invasive breast cancer: prognostic implications. *Clinical Cancer Res.*, **1**, (in press).

WABER PG, CHEN J AND NISEN PD. (1993). Infrequency of MDM2 gene amplification in pediatric solid tumors and lack of association with p53 mutations in adult squamous cell carcinomas. *Cancer Res.*, **53**, 6028–6030.